# Validation of END-of-life ScorING-system to identify the dying patient: a prospective analysis

**DOI:** 10.1186/s12871-020-00979-y

**Published:** 2020-03-09

**Authors:** Gianluca Villa, Timothy Amass, Rosa Giua, Iacopo Lanini, Cosimo Chelazzi, Lorenzo Tofani, Rory McFadden, A. Raffaele De Gaudio, Sean OMahony, Mitchell M. Levy, Stefano Romagnoli

**Affiliations:** 1 Section of Anesthesiology, Intensive Care and Pain Therapy, Departmnt of Health Sciences, University of Florence, Florence, Italy; 2 Department of Anesthesia and Intensive Care, Azienda Ospedaliero-Universitaria Careggi, Largo Brambilla,3, 50134 Florence, Italy; 3 Department of Medicine, Division of Pulmonary Critical Care & Sleep, Brown University, Providence, RI USA; 4 Department of Internal Medicine, Palliative Medicine Section, Rush University Medical Center, Chicago, IL USA

**Keywords:** End of life, Scoring system, palliative care, intensive care unit

## Abstract

**Background:**

The “END-of-Life ScorING-System” (ENDING-S) was previously developed to identify patients at high-risk of dying in the ICU and to facilitate a practical integration between palliative and intensive care. The aim of this study is to prospectively validate ENDING-S in a cohort of long-term critical care patients.

**Materials and methods:**

Adult long-term ICU patients (with a length-of-stay> 4 days) were considered for this prospective multicenter observational study. ENDING-S and SOFA score were calculated daily and evaluated against the patient’s ICU outcome. The predictive properties were evaluated through a receiver operating characteristic (ROC) analysis.

**Results:**

Two hundred twenty patients were enrolled for this study. Among these, 21.46% died during the ICU stay. ENDING-S correctly predicted the ICU outcome in 71.4% of patients. Sensitivity, specificity, positive and negative predictive values associated with the previously identified ENDING-S cut-off of 11.5 were 68.1, 72.3, 60 and 89.3%, respectively. ROC-AUC for outcome prediction was 0.79 for ENDING-S and 0.88 for SOFA in this cohort.

**Conclusions:**

ENDING-S, while not as accurately as in the pilot study, demonstrated acceptable discrimination properties in identifying long-term ICU patients at very high-risk of dying. ENDING-S may be a useful tool aimed at facilitating a practical integration between palliative, end-of-life and intensive care.

**Trial registration:**

Clinicaltrials.gov Identifier: NCT02875912; First registration August 4, 2016.

## Background

The advanced technological treatments available in the intensive care unit (ICU) aim at managing acute illness and at the same time supporting multiorgan failure [1]. Considering an average mortality rate of 20% among critical care patients, only 80% of ICU patients really benefit from these highly expensive treatments [1]; furthermore, a not irrelevant percentage of ICU survivors will die in any case before hospital discharge [2]. Given the likelihood of morbidity or mortality, palliative and end-of-life care should be routinely considered in the ICU [3]. As such, the World Health Organization has described palliative care as “an approach that improves the quality of life of patients and their families facing the problems associated with life-threatening illness” [4]. The physician should be thus aware of the patient and family needs to improve the management of physical, psychological and spiritual symptoms. Additionally, being aware of the value of palliative care services to help meet the families’ and patients’ needs and align therapy to the prognosis of the patient balanced with their preferences and values [4, 5].

Beyond those clearly at the final stages of irreversible diseases (i.e. end-of-life), all patients admitted in the ICU require an early integration between a comprehensive palliative approach and intensive care treatment [4, 6]. The probability of dying of each critical care patient should be evaluated early on at the ICU admission and continuously reassessed during the entire ICU length of stay; the amount of palliative care treatments should thus be integrated accordingly [4].

Although excellent scoring systems are available in the ICU for prognostic and clinical monitoring purposes (e.g. Acute Physiologic Assessment and Chronic Health Evaluation, APACHE, and Sequential Organ Failure Assessment score, SOFA [7]), an accurate identification of end-of-life patients in the ICU is still cumbersome [8–10]. As example, the usefulness of several scores (e.g. the APACHE score) is mainly validated at the ICU admission, when the patients’ responsiveness to intensive care treatments is not clear yet. Other scoring systems (e.g. the SOFA score) are well validated to monitor the organ dysfunctions over time in the ICU; nevertheless, the requirement of biochemical data for the scoring calculation may limit their routine use/daily application [7]. Finally, the requirement for, and to what degree, palliative care should be integrated with intensive care for all ICU patients is often not objectively defined and instead determined by individual physician’s perspective [11, 12].

The “END-of-Life ScorING-System” (ENDING-S) was previously developed to: 1) identify patients at very high risk of dying in the ICU and 2) facilitate a practical integration between palliative, end-of-life and intensive care treatments [11]. In a pilot study, ENDING-S presented acceptable calibration and discrimination properties in identifying patients at very high risk of dying in the ICU, with a receiver operating characteristic-area under the curve (ROC-AUC) analysis equal to 0.98 (95%CI, 0.97 to 1) and agreement between the predicted probability and the observed frequency of death in the ICU (*p* > 0.05 at Hosmer-Lemeshow test) were preliminarily observed [11].

The aim of this observational study is to prospectively validate ENDING-S in a cohort of critical care patients with an ICU length of stay longer than 4 days.

## Methods

This observational prospective study was performed in three ICUs: Rhode Island Hospital’s medical ICU (Providence RI), Rush Medical Center’s medical ICU (Chicago IL) and Azienda Ospedaliera Universitaria Careggi’s surgical and medical ICU (Florence Italy). The institutional review boards of each center reviewed and approved the protocol (clinicaltrials.gov Identifier: NCT02875912; first retrospective registration: August 4, 2016). Written consent for analysis and publication of clinical data was obtained from all consentable patients. If the patient was not able to sign consent forms at the study enrollment, permission for analysis and publication of clinical data was obtained from a surrogate or waived in accordance with local ethics committee.

All adult patients admitted in the ICU from September 2015 to March 2017 were considered eligible for the study. Patients admitted to the ICU for end-of-life care were excluded. In order to consider only the long-term ICU patients, those with an ICU length of stay shorter than 4 days were excluded from the analysis. Data abstraction forms were prospectively completed for all eligible patients. In particular, ENDING-S and SOFA scores were calculated daily from the ICU day 4 to the ICU discharge for each enrolled patient. In accordance with the previous paper [1], ENDING-S score was calculated as:

$$ \mathrm{ENDING}-\mathrm{S}=\left(7.25\bullet \mathrm{Days}\ \mathrm{of}\ \mathrm{MV}/\mathrm{ICU}\ \mathrm{LoS}\right)+\left(10.45\bullet \mathrm{Days}\ \mathrm{of}\ \mathrm{Vasoactive}\ \mathrm{drugs}/\mathrm{ICU}\ \mathrm{LoS}\right)+\left(3\bullet \mathrm{Sepsis}\right)+\left(0.3\bullet \mathrm{ICU}\ \mathrm{LoS}\right). $$

where Days of MV/ICU LoS expresses the ratio between the current days in which the patient requires mechanical ventilation (MV) and the current ICU length of stay (LoS, quantitative variable), Days of Vasoactive drugs/ICU LoS expresses the ratio between the current days in which the patient requires vasoactive drugs and the current ICU length of stay (quantitative variable), Sepsis expresses a septic condition (dichotomous variable: 1 if currently affected, otherwise 0), ICU LoS expresses the current ICU length of stay (quantitative variable).

The ICU patients’ management has not changed according to the value of ENDING-S observed.

According to the patients’ outcome, the enrolled population was divided into two groups, either “survived” or “died”. Death in the ICU being used as a surrogate to identify those ICU patients that had been at the end of their life prior to death. For “died” patients, maximization of palliative care and end-of-life care are thus certainly required. For “survived” patients, a certain form of palliative care is required according to the patients’ multidimensional evaluation and expected prognosis, but very unlikely these patients require end-of-life care.

The association between the daily values of ENDING-S and patients’ outcome at the ICU discharge was tested through a logistic regression analysis (OR, 95% CI). The ENDING-S predictive properties were evaluated with a ROC analysis. The effectiveness of ENDING-S cut-off 11.5 was previously validated for prediction of death during the ICU stay. The positive predictive value (PPV), negative predictive values (NPV), sensitivity and specificity of ENDING-s were calculated.

Similarly, the association between the daily values of SOFA score and patients’ outcome was tested through a logistic regression analysis. The effectiveness of SOFA score was assessed for prediction of death during the ICU stay. In the absence of a specific cut-off point, only a ROC analysis was performed for this scoring system.

Continuous parameters observed in the population are reported as median [interquartile range] or mean ± standard deviation (SD), where appropriate; dichotomous parameters are expressed as crude number and percentage. A *p*-value of 0.05 has been considered for statistical significance.

Data was analyzed using STATA 9.1 software (STATA corp, 490, Lakeway Drive College Station, 77,845, Texas, US).

## Results

**Fig. 1:**
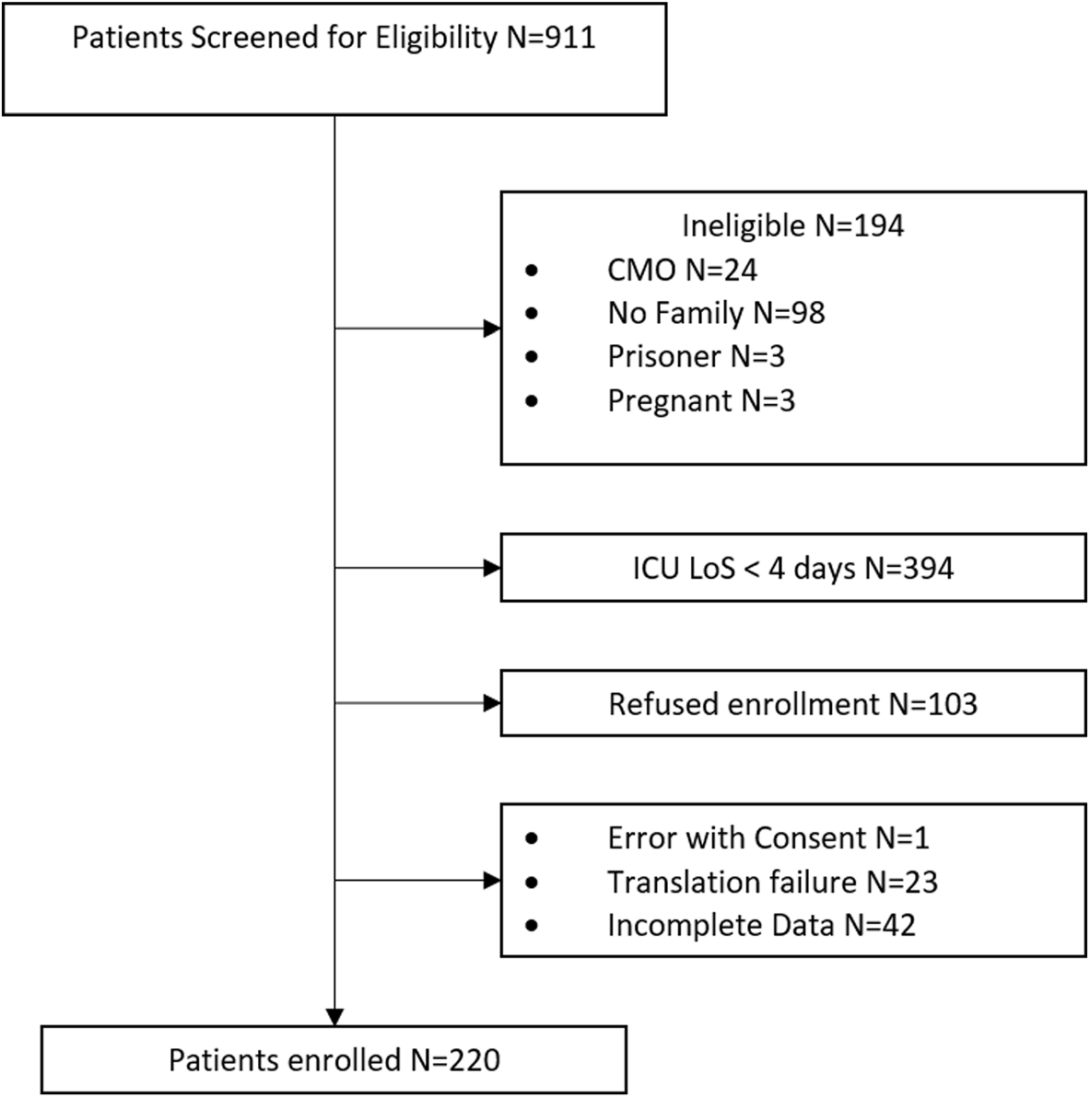
The enrollment process. Over the entire population potentially eligible for this prospective study, 194 patients were excluded because admitted in the ICU for comfort measure only (CMO), for lack of family members (required for the qualitative analysis of the study, data not presented), or because pregnant or prisoner. In order to consider only “long term” ICU patients, those with an ICU length of stay (LoS) < 4 days were excluded. Finally, 103 patients refused to be enrolled in this observational study, forms were not completed for 43 patients (1 consent form and 42 clinical data forms) and 23 patients were excluded because not English or Italian native speaking

Nine hundred and eleven patients were prospectively screened for this multicenter study. Among these, 220 patients had an ICU length of stay longer than 4 days and thus prospectively enrolled and considered for the analysis. The enrollment procedures are reported in Fig. 1.

Among the enrolled patients, 21.46% (47/220) died during the ICU stay, while 78.64% (173/220) survived and were discharged from the ICU. The patients’ characteristics, for both survivor and death groups, are reported in Table 1.

**Table 1:** Patients’ characteristics at the ICU admission and at discharge from the ICU

	All (***n*** = 220)	Death (*n* = 47)	Survivor (*n* = 173)	*p*
**Age** (yrs)	64.5 ± 16.8	66.1 ± 17.1	64.1 ± 16.6	0.12
**Sex**				0.41
*Male*	128 (58.1%)	30 (63.8%)	98 (56.6%)	
*Female*	92 (41.9%)	17 (36.2%)	75 (43.4%)	
**Race**				0.04
*White*	181 (82.3%)	34 (72.3%)	147 (84.9%)	
*Black or African American*	12 (5.4%)	6 (12.8%)	6 (3.5%)	
*Other*	27 (12.3%)	7 (14.9%)	20 (11.6%)	
**Country of birth**				0.06
*Italy*	119 (54%)	19 (38.4%)	101 (58.3%)	
*USA*	76 (34.3%)	22 (46.8%)	54 (31.1%)	
*Other*	1 (0.4%)	0 (0%)	1 (0.6%)	
**Admission source**				0.15
*Home*	54 (24.3%)	14 (29.8%)	40 (23.1%)	
*Nursing home/Skilled nursing facility*	13 (5.9%)	5 (10.6%)	8 (4.6%)	
*Acute care facility/Outside hospital*	153 (69.8%)	28 (59.6%)	125 (72.3%)	
**SOFA score at ICU admission**	5.4 ± 3.4	8.6 ± 3.6	4.8 ± 2.9	< 0.01
**Vasoactive medication requirements at ICU admission**	30 (13.6%)	10 (21.3%)	20 (11.6%)	0.09
**PaO2/FiO2 ≤ 300 at ICU admission**	44 (20%)	17 (36.2%)	27 (15.6%)	< 0.01
**Serum creatinine ≥ 2 mg/dl at ICU admission**	23 (10.5%)	10 (21.3%)	13 (7.5%)	0.01
**Serum bilirubin ≥ 2 mg/dl at ICU admission**	5 (2.3%)	3 (6.4%)	2 (1.1%)	0.07
**Length of ICU stay (days)**	10.3 ± 6.6	11.6 ± 6.4	9.9 ± 6.9	0.07
**Status change in DNR/DNI/CMO**				< 0.01
Yes	55 (25.7%)	41 (87.2%)	14 (8.1%)	
No	165 (74.3%)	6 (12.8%)	159 (91.9%)	
**After discharge**				NA
**Another hospital floor**			155 (89.6%)	
**Nursing home**			2 (1.2%)	
**Long-term ventilator assist facility**			5 (2.9%)	
**Home**			3 (1.7%)	
**Other**			8 (4.6%)	

Abbreviations: *DNI/DNR/CMO* Do not intubate/Do not resuscitate/Comfort measure only

Among patients who died, 32 of 47 (68.09%) had ENDING-S values higher than 11.5 (true positive), while the remaining 15 (31.91%) had at least an ENDING-S < 11.5 during the ICU stay (false negative). However, among patients who survived to ICU discharge, 125 of 173 (72.25%) had ENDING-S values smaller than 11.5 (true negative), while the remaining 48 (27.75%) had at least an ENDING-S ≥ 11.5 during the ICU stay (false positive). Given these characteristics, ENDING-S correctly predicted for 157 patients (157/220, 71.4 95%CI [0.65–0.77] and was statistically associated with the patients’ ICU outcome, *p* < 0.0001, OR 1.182, 95%CI [1.115–1.253].

**Fig. 2:**
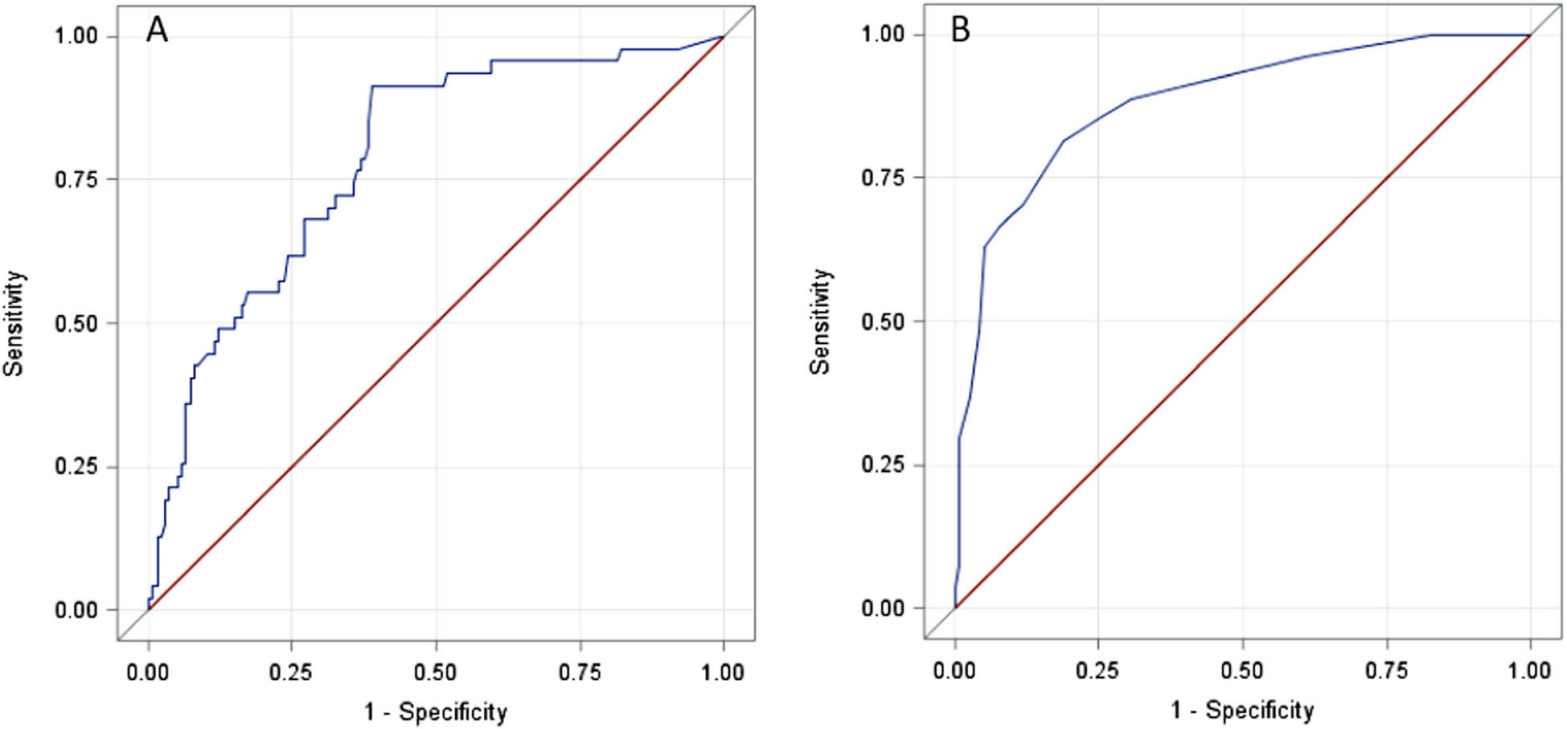
Likelihood of ICU Death. ROC curves for patients’ outcome discrimination for both ENDING-S (Panel **a**, ROC-AUC 0.79) and SOFA score (Panel **b**, ROC-AUC 0.88)

Sensitivity and specificity associated with ENDING-S cut-off of 11.5 were 68.09 and 72.25%, respectively; positive and negative predictive values were 60 and 89.28%, respectively. ROC-AUC of 0.79 was found for ENDING-S in this validation set (Fig. 2, Panel a), 95% CI [0.71–0.86].

Considering daily values of SOFA score both for survived and not survived patients, a mean SOFA score of 8.4 ± 4.1 was observed for patients who died, while 4 ± 2.6 was observed for patients who survived at the ICU discharge. A ROC-AUC of 0.88 (95%CI 0.81–0.96) was found for daily values of SOFA score in predicting ICU death (Fig. 2, Panel b).

## Discussion

In this observational study, the previously defined ENDING-score was prospectively tested in a cohort of critical care patients with an ICU length of stay longer than 4 days in order to validate its discriminative effect in identifying patient at very high risk of dying in the ICU.

The comparison between the ICU outcome observed for every enrolled patient and that expected according to the ENDING-S cut-off of 11.5 reveals a direct association between ENDING-S and patients’ ICU outcome *p* < 0.0001). In particular, every incremental increase in ENDING-S value increased the OR estimate of death in the ICU by 1.18.

Compared with discrimination properties showed by ENDING-S in the pilot study, less efficient characteristics were observed in this prospective validation. Indeed, a ROC-AUC of 0.98 (95%CI, 0.97–1.00) was observed in the calibration set of the pilot study and further confirmed in the internal validation performed in the same study (ROC-AUC 0.98; 95%CI 0.96–1.00). On the other hand, a ROC-AUC of 0.79 (95%CI 0.71–0.86) was observed in this prospective external validation. This effect was expected considering that a cross-validation test was applied in the pilot study to internally validate the model and to assess the predictive properties of ENDING-S. Although statistically correct, the use of an internal validation might have optimistically confirmed the preliminary results, overestimating the ENDING-s performance characteristics. Nevertheless, a ROC-AUC equal to 0.79 is still sufficient to confirm the acceptable discrimination properties of ENDING-S in identifying patients at very high risk of dying in the ICU.

Similar to the ROC-AUC, the sensitivity and specificity values previously observed in the pilot study are reduced in this external validation study [11].

The identification of end-of-life patients is quintessential for an adequate integration between qualitative and intensive care treatments during the ICU stay [13, 14]. Notably, palliative care should not be considered as an alternative for the intensive care in these patients; this care should instead be concomitantly made available for the patients and their family early on from the ICU admission [15]. The importance of palliative care with respect to intensive care should be proportional with the probability that the patient is at very high risk of dying in the ICU and in accordance with the specific patient and family needs.

Identification of the patient at very high risk of dying is not the only limitation for palliative care integration in the ICU [16]. Indeed, the “relative amount” of palliative care that should be considered adequate for a specific patient in a specific moment during the ICU stay is difficult to define. Most of data in literature show that the management of palliative and end-of-life care is still determined by the physician’s subjective experiences, religion, level of expertise and other nonobjective and un-quantifiable variables [1, 15, 17–19]. Used beside other tools and comprehensive clinical evaluations, this objective tool, able to accurately identify dying patients and to suggest the adequate ratio between qualitative and intensive care for those patients at very high-risk of dying in the ICU, might help the physician appropriately integrate palliative care in the ICU [9].

**Fig. 3:**
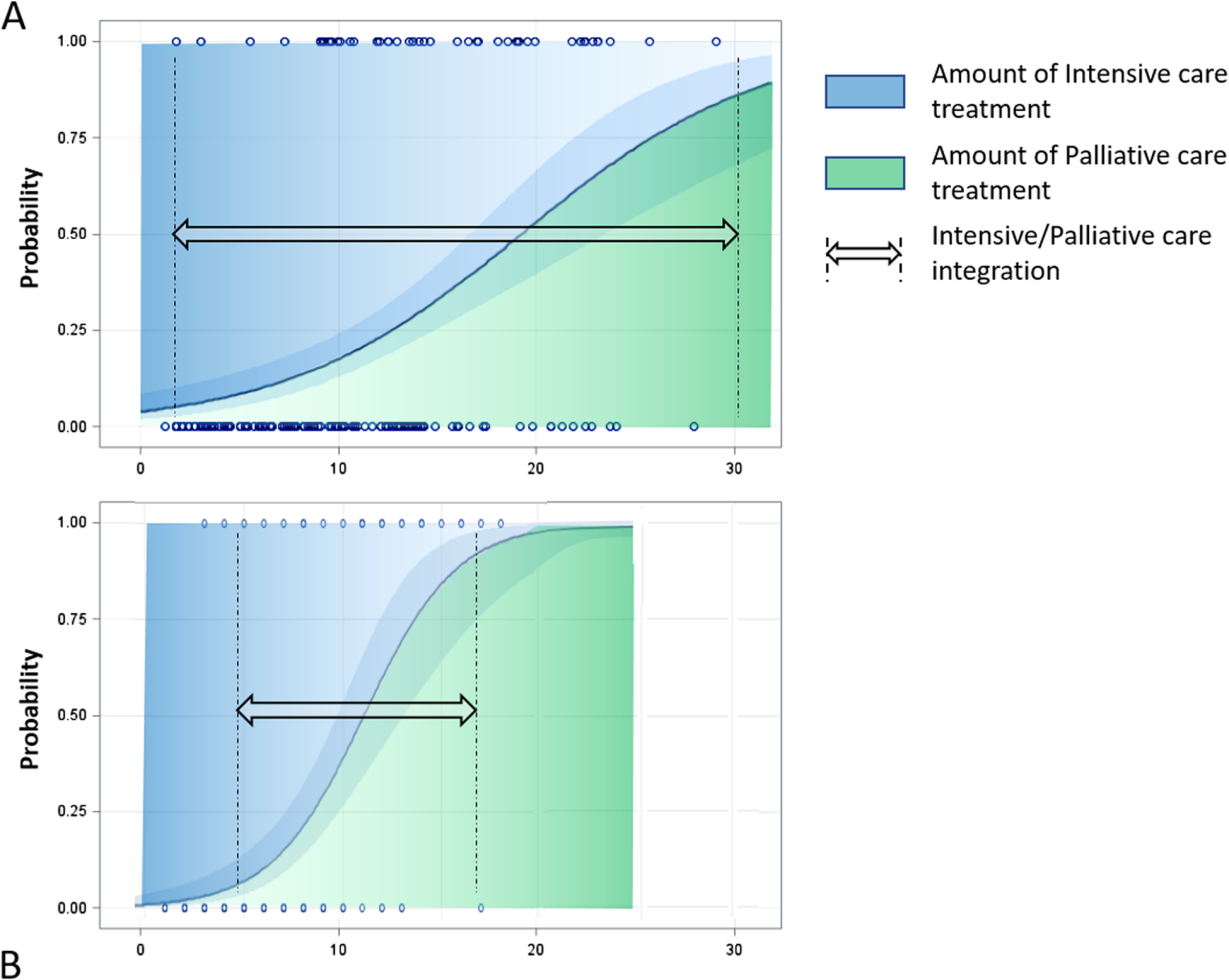
Probability of ICU Death as compared to increasing values of ENDING-S (panel **a**) and SOFA score (panel **b**). For each patient, the higher the ENDING-s or SOFA score, the higher the probability of ICU death, the higher the amount of palliative care interventions (in green) that should be integrated with intensive care treatment (in blue). Palliative care and intensive care should not be mutually exclusive; they should instead integrate each-other during the entire course of the patient’s disease from the diagnosis and the initial organ dysfunction to the occurrence of multiorgan failure and end-of-life condition (within the dashed line). An appropriate scoring system should be characterized by a slope in score/outcome probability able to promote intensive care and palliative care integration continuously, and across different levels of patient’s severity

Assuming that the progression toward end-of-life should be characterized by an increasing presence of palliative care in the global management of the patient, the probability of being at very high risk of death in the ICU can be used as an indicator of the percentage need of palliative and mainly end-of-life care integration. Treatments based on patients’ and families’ needs (i.e. targeted on communication, psychological, social personal and/or spiritual well-being), should be progressively prioritized within the efforts of the health care team. In these terms, evaluating the ENDING-S and SOFA score might also be potentially useful for guiding palliative care integration in the ICU (Fig. 3).

While SOFA does outperform ENDING-S, ENDING-S may still be considered clinically useful when compared to a SOFA score in two ways. First, the negative predictive value of ENDING-S of 89.28% suggests that an ENDING-S score less than 11.5 is clinically valuable in aiding clinicians in identifying patients that are likely to survive. Combining this with the fact that an ENDING-S score does not require any laboratory data as the SOFA score does, ENDING-S can be calculated every day, easily, on each patient. SOFA score requires laboratory data which was often missing in this cohort limiting the usefulness of a SOFA score in providing daily prognostic information.

Another important difference between ENDING-S and SOFA score is in the variation during the ICU length of stay of clinically stable patients who spend their end-of-life period in the ICU before dying. Although in critical condition, several patients have high and unchanged values of SOFA score over time, specifically before dying. Thus, a prognostic tool only based on SOFA score would not change, failing to alert the physician to the increasing amount of palliative and end-of-life care required for that patient. On the other hand, as ENDING-S is influenced by the progression of days spent in the ICU, it increases over time even in absence of clinical changes. This suggests that, by virtue of staying in the ICU, patients could require a progressively increasing amount of qualitative care over time.

There are several limitations in this study; among these, the use of ICU mortality as a surrogate to identify end-of-life is the most important. The aim of ENDING-S is to identify those patients likely being at very high risk of dying in the ICU and suggest the physician to appropriately consider palliative care integration for these patients. Interestingly, an objective definition of end-of-life status is still lacking in clinical practice, even for patients who are not hospitalized [8]. For this reason, as in other papers [20, 21], the outcome observed at the ICU discharge has been used as a surrogate for end-of-life condition for patients both enrolled in the pilot study and for those enrolled in this prospective validation. Notably, despite death in the ICU certainly being associated with the end-of-life of patients, ICU survival does not necessarily exclude the end-of-life condition or the requirement of palliative care [22]. Unfortunately, a systematic follow-up for patients discharged from the ICU is lacking in this study.

Another important conceptual limitation refers to the exclusive use of the patient’s prognosis to guide the palliative care/intensive care integration. Several authors agree that palliative care should be based on needs of patients and their family instead of being exclusively based on patients’ prognosis. A comprehensive qualitative evaluation should be integrated on ENDING-S parameters to further improve its role in guiding palliative care. Issues such as patients and family needs, perception of the disease, communication between family members and with health care providers should be considered and analyzed with this aim.

## Conclusion

Although values observed in the pilot study resulted in a slightly overestimated prediction model, acceptable discrimination properties have been demonstrated for ENDING-S in identifying patients at very high risk of dying with an ICU stay longer than 4 days. Although SOFA score was more effective and accurate in predicting patients’ death in the ICU, the ENDING-S score offers the benefit of not requiring laboratory data, a strong negative predictive value for ICU death, and daily changes by including length of stay. These factors may allow ENDING-S to be meaningfully integrated into daily practice and aid in the integration between palliative, end-of-life and intensive care. Nevertheless, further prospective validations and comparisons between EDNING-S and other standardized score are necessarily to better characterize the clinical application of this tool.

## Data Availability

The datasets analysed during the current study are available from the corresponding author on reasonable request.
